# A Strange Coincidence

**Published:** 1890-03

**Authors:** 


### A STRANGE COINCIDENCE.

On the 17th day of April last, Mrs. Carroll, of 51 North Sheldon street, Chicago, Ill., received a letter from a relative in this city informing her that an old schoolmate here was in the last stage of consumption and would live but a few days longer. The news prostrated the lady and for hours she seemed to be unconscious, though whispering constantly "Katy must not die; give her carbonic acid gas." During the day a friend of the family, a medium, called and on entering the room exclaimed, "Why Mrs. C., you have company to-day. I see an old German standing by your bedside trying to tell you about some medicine." Almost immediately the woman was entranced and called for pencil and paper and began to write in German. No one present could read a word of it, and it was taken to a German druggist near by who translated it, declaring it must have been written by a German chemist, as it was a formula for making and administering "carbonic acid gas." The formula was entirely new to him but correct in every particular.

Mrs. Carroll was so impressed with this, to her, strange manifestation, that she immediately sent the formula to her friend in this city, relating the circumstances minutely and urging her friend to try it. She did so and began to improve at once, and on June 1st was able to ride and walk out daily, and at the present time is attending to her household duties.

The druggist sent a statement of the affair to the *Medical Record*, and received in reply a statement from the editor of that journal, that on the 7th day of April Dr. Hugo Nieber of Berlin, began treating consumption in a hospital of that city; and that in the twelve cases reported by him, all had been favorably affected by the treatment, and seven were in a fair way to a final recovery. The formula given through the medium for preparing and administering the gas was identical with the one discovered the same day by Dr. Nieber and published in the *Berlin Medical Journal* of June 2nd.—*Rochester Republican.*

